# Ecological implications of metabolic compensation at low temperatures in salamanders

**DOI:** 10.7717/peerj.2072

**Published:** 2016-05-24

**Authors:** Alessandro Catenazzi

**Affiliations:** Department of Zoology, Southern Illinois University Carbondale, Carbondale, IL, USA

**Keywords:** Heart rate, Amphibian, Global warming, Performance breadth, Conservation, Body temperature

## Abstract

Global warming is influencing the biology of the world’s biota. Temperature increases are occurring at a faster pace than that experienced by organisms in their evolutionary histories, limiting the organisms’ response to new conditions. Mechanistic models that include physiological traits can help predict species’ responses to warming. Changes in metabolism at high temperatures are often examined; yet many species are behaviorally shielded from high temperatures. Salamanders generally favor cold temperatures and are one of few groups of metazoans to be most species-rich in temperate regions. I examined variation in body temperature, behavioral activity, and temperature dependence of resting heart rate, used as a proxy for standard metabolic rate, in fire salamanders (*Salamandra salamandra*). Over 26 years, I found that salamanders are behaviorally active at temperatures as low as 1 °C, and aestivate at temperatures above 16 °C. Infrared thermography indicates limited thermoregulation opportunities for these nocturnal amphibians. Temperature affects resting heart rate, causing metabolic depression above 11 °C, and metabolic compensation below 8 °C: heart rate at 3 °C is 224% the expected heart rate. Thus, salamanders operating at low temperatures during periods of peak behavioral activity are able to maintain a higher metabolic rate than the rate expected in absence of compensation. This compensatory mechanism has important ecological implications, because it increases estimated seasonal heart rates. Increased heart rate, and thus metabolism, will require higher caloric intake for field-active salamanders. Thus, it is important to consider a species performance breadth over the entire temperature range, and particularly low temperatures that are ecologically relevant for cold tolerant species such as salamanders.

## Introduction

Ongoing climate change is influencing the biology and distribution of living organisms (Beebee, 1995; Walther et al., 2002). Climatic changes are occurring at a much faster pace than that experienced by living organisms in their evolutionary histories (Beaumont et al., 2011), and there is growing concern than many species will be unable to overcome the challenges of a quickly changing climate (Parmesan, 2006). An organism’s potential response to climate change includes physiological, behavioral, ecological and evolutionary responses (Huey et al., 2012), but it is generally understood to be hindered by anthropogenic stressors. Therefore, many organisms are projected to become vulnerable, particularly species that lack physiological plasticity and behavioral responses allowing them to mitigate the effects of future climatic changes (Somero, 2010).

It has been proposed that physiological tolerances and constraints can help predict vulnerability of organisms to climate change (Deutsch et al., 2008; Dillon, Wang & Huey, 2010; Fuller et al., 2010; Huey et al., 2012). Mechanistic models for species responses to warming and species distribution models increasingly include such components (Austin et al., 2009; Kearney & Porter, 2004), supplementing bioclimatic niche envelope approaches (Araújo & Peterson, 2012; Kearney, Wintle & Porter, 2010). However, for these physiological constraints to be meaningful we need ways to interpret the biological relevance of variation in performance (Fuller et al., 2010; Magozzi & Calosi, 2015), and specifically, its contribution to organism’s fitness (Asbury & Angilletta, 2010). We also need to better document variation in body temperature, including for animals whose body temperature is often assumed to be equal or similar to air or substrate temperature.

Thermal performance curves describe the effects of temperature on the rate of biological processes such as locomotion, growth and reproduction (Huey & Stevenson, 1979). These curves share the important property of responding rapidly and reversibly to changes in temperature (Angilletta, 2009). Thermal performance curves are typically bell-shaped, with a slowly rising and linear shape below the thermal optimum, a rounded peak, and a precipitous drop in performance as temperature approaches the critical maximum tolerated by the organism. We can consider the linear and rising component of the curve as a good approximation of expected performance, because over this range of temperatures the organism is most likely to evade behavioral and physiological costs associated with lower or higher temperatures. We can then calculate “null” expected performance at lower and higher temperatures, i.e., the level of performance that would occur in the absence of thermoregulatory costs. This conceptual approach is beneficial because it allows us to better understand the vulnerability of organisms to climate change (Bernardo & Spotila, 2006).

Salamanders are well known for favoring cool temperatures (Brattstrom, 1963; Feder & Lynch, 1982; Spotila, 1972) and for tolerating freezing. In 1795, the Italian physiologist Spallanzani constrained different vertebrates to freezing temperatures, and found that salamanders were the most freezing-tolerant of all organisms he tested (Spallanzani, 1994). The salamander tree of life also attests to their preference for cold temperatures: salamanders are one of few groups of terrestrial metazoans to be evolutionarily most clade- and species-rich in temperate regions (Duellman, 1999; Vieites, Min & Wake, 2007). Therefore, warming climates do not bode well for salamanders, and salamanders might be the terrestrial vertebrates most vulnerable to climate warming. Studies from salamander-rich Appalachia have predicted (Milanovich et al., 2010) or reported population declines (Caruso & Lips, 2013) and reductions in body size putatively associated with increases in average air temperature (Caruso et al., 2014). Increased energetic demands associated with higher temperatures may force salamanders to allocate more energy to maintenance and reproduction, and less energy to growth. Although salamanders may be able to behaviorally evade warm temperatures (for example during summer, by retreating to underground burrows), they may be at high risk from chronic exposure to warming during periods of above ground activity, because of the greater metabolic costs they will incur.

Mitigation of high and low temperatures involves different behavioral and physiological mechanisms and requires different sets of adaptations. Most salamanders are nocturnal and inhabit permanently moist environments, limiting opportunities for thermoregulation to heat exchanges through conduction (Feder, 1983). In salamanders, behavioral mitigations of high temperature may include evasion, immersion in water followed by evaporative cooling, and retreating to underground habitats. Physiological processes include acclimation of metabolic rate (Feder, 1982), acclimation of critical thermal maximum (Hutchison & Maness, 1979), and expression of heat shock proteins (Near et al., 1990). Tolerance to low temperatures, in addition to acclimation responses, involves the use of cryoprotectants such as glucose and glycerol (Storey & Storey, 1986).

My study had four goals. First, I aimed to document the extent to which a temperate salamander species is behaviorally active at low temperatures. Although behavioral activity at low temperatures has been reported, body temperatures of field-active salamanders have not been measured. Second, I aimed at determining the temperature dependence of resting heart rate, used here as a proxy for standard metabolic rate (Green, 2011). Width of the performance breadth and degree of thermal sensitivity are important metrics for predicting species’ responses to climate warming. Third, I calculated the extent to which salamanders’ heart rates deviate from the “null” rate expected in absence of thermoregulatory costs. The expectation is that salamanders, as reported in previous publications (Bernardo & Spotila, 2006), display metabolic depression at high temperatures, but researchers have rarely examined metabolic responses at temperatures below 5 °C. Lastly, I investigated the ecological implications of metabolic compensation at low temperatures. In many places, climate warming will produce shorter and warmer winters that will increase the time cold-tolerant organisms are active at near-freezing temperatures. Here, I show that such metabolic compensation has important implications for predicting species’ vulnerabilities to climate change.

## Materials and Methods

### Study site and organism

**Figure 1:**
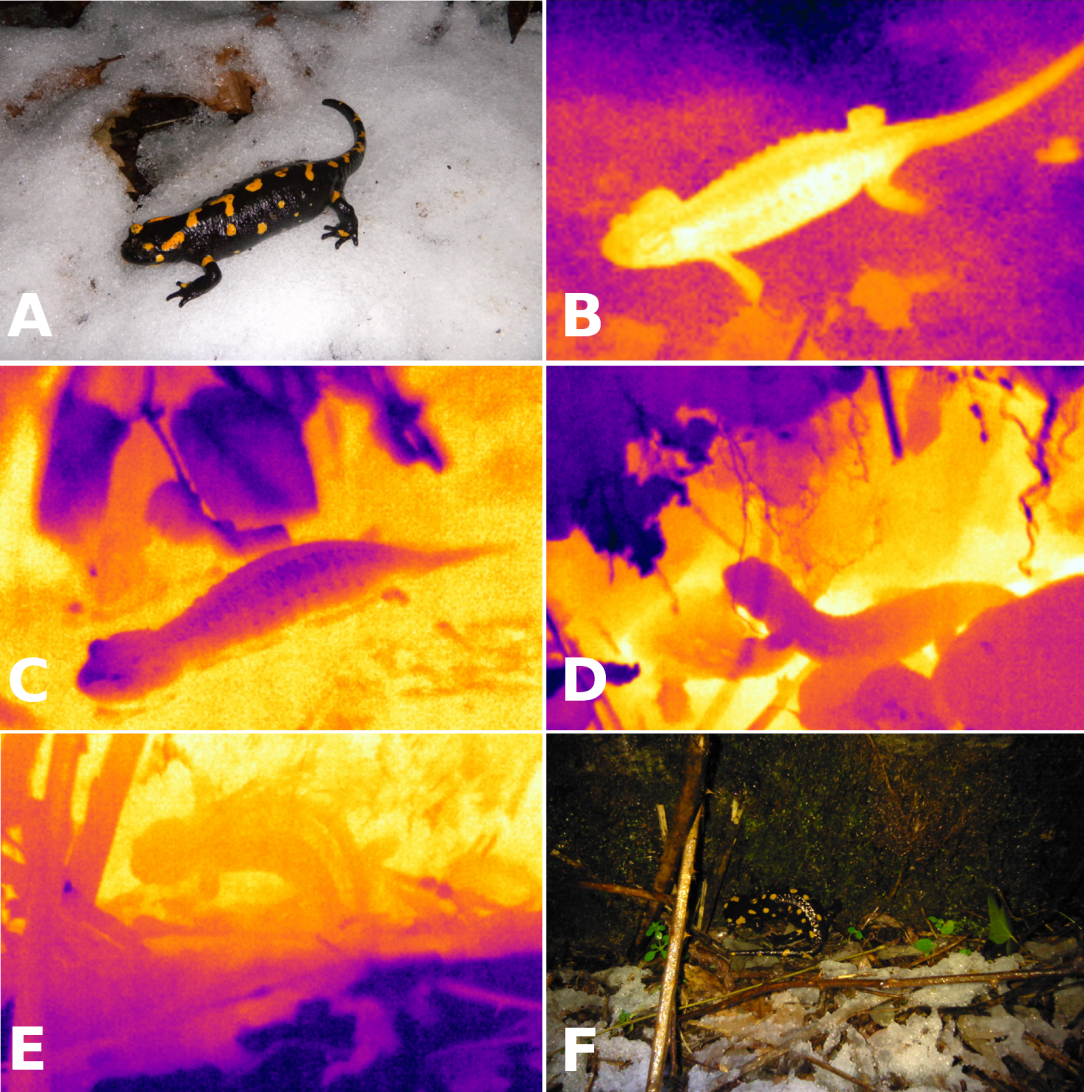
Visible light and infrared images of salamanders. Visible light (A) and infrared (B) images of a fire salamander (*Salamandra salamandra*, *T*_*b*_ = 3.2 °C) active on a layer of snow (*T*_*s*_ = − 0.3 °C) on 2 January 2014 (*T*_*a*_ = 2.0 °C). Infrared images illustrating avoidance of near-freezing temperatures: immersion in spring water (C; *T*_*b*_ = 7.5 °C, water in yellow is at 8.9 °C, leaves in dark blue are at 6.4 °C), retreat to underground burrows (D; two salamanders with *T*_*b*_ of 6.4 °C and 5.5 °C are entering the same burrow, the soil is in yellow at 7.2 °C whereas the leaf litter is in dark blue at 3.9 °C) and contact with not-so-cold rocks (E, F: *T*_*b*_ = 1.7 °C, bedrock in yellow is at 2.7 °C, snow in dark blue is at −1.0 °C).

I studied a population of fire salamanders (*Salamandra salamandra*) along a forested creek in the Lepontine Alps of southern Switzerland (46.1595°N, 9.0028°E; 295 m.a.s.l., Camorino, Ticino). European salamanders show wide variation in use of winter torpor. In western and central Europe, fire salamanders are active during spring, summer and fall, with winter torpor or brumation limiting their activity during winter (Klewen, 1985; Thiesmeier, 2004; Thiesmeier & Grossenbacher, 2004). A contrasting pattern is found in the Iberian Peninsula and around the Mediterranean, where salamanders aestivate but do not hibernate (Bruno, 1973; Rebelo & Leclair, 2003). Throughout their range, fire salamanders are known to be remarkably tolerant of cold temperatures (Bruno, 1973; Grossenbacher, 1988; Spallanzani, 1994), and salamanders have been found walking on snow or active above ground at near-freezing temperatures (Figs. 1A and 1B).

I visited the same 700 m^2^ area 322 times over a period of 26 years: 20 times from October 1989 to April 1990, 278 times from September 1992 to October 1998, 3 times from October to December 1999, once in December 2011, twice from September to December 2012, and 18 times from December 2013 to January 2016. I surveyed the entire area and counted all field-active salamanders, i.e., all salamanders that were observed on the surface outside of burrows and above the leaf litter, and avoided any habitat disturbance that could affect salamander occurrence during posterior visits (i.e., no efforts were made to turn over rocks, remove leaf litter, etc. in search of salamanders). The study area has little understory vegetation, but varying amounts of leaf litter might negatively affect detection probability during fall and early winter, when I observed the highest numbers of field-active salamanders.

Dates of surveys were chosen haphazardly and temperature and rainfall during surveys overall are representative of the local climatic conditions. However, all surveys since 2011 included at least one December visit, because during each of the seven reproductive seasons from 1992 to 1998 (*n* = 278 visits), the highest number of field-active salamanders was observed during a rainy December night. I averaged monthly numbers of field-active salamanders by dividing the total number of salamanders by the number of visits for each month.

### Temperature measurements

Air temperature (1.5 m above ground) and moisture were recorded at the study site during each visit from 1989 to 2016 with a digital thermo-hygrometer. From December 2013 to January 2016, I used a handheld infrared camera (E60, Flir Systems AB) to measure body (*T*_*b*_) and substrate temperatures of field-active salamanders. The infrared camera operates in the 7.5–13 µm spectral range, and has a spatial resolution of 1.36 mrad and an image resolution of 320 × 5240 pixels. To obtain corrected values of *T*_*b*_ by compensating for the effects of different radiation sources, the following settings were adjusted for thermographic analyses: emissivity, distance between salamander and camera, air temperature, air humidity, and reflected temperature (measured by using corrugated aluminum foil). Emissivity for amphibian skin generally varies from 0.95 to 0.97 (Carroll, Irwin & Green, 2005; Gates, 1980; Rowley & Alford, 2007), but varying the value in my analyses did not change *T*_*b*_ in excess of 0.1 °C.

Thermal imaging accurately measured body temperature of field-active and indoor salamanders. I compared temperatures obtained with the infrared camera (*T*_*IR*_) with cloacal temperatures (*T*_*CL*_) obtained with a quick-reading thermometer (Miller-Weber; precision 0.2 °C) for 79 salamanders in the field and of 41 salamanders indoors and whose temperatures were measured in the same room and under the same conditions used for the heart rate measurements (see below). Under both circumstances, infrared readings were on average within 0.2 °C of the cloacal measurement, and mean differences in temperatures (Δ_*t*_ = *T*_*CL*_ − *T*_*IR*_) were Δ_*t*_ = 0.160 ± 0.005 °C outdoors (tested range *T*_*CL*_ = 0.8–5.9 °C), and Δ_*t*_ = 0.002 ± 0.004 °C indoors (tested range *T*_*CL*_ = 2.8–20.2 °C). These differences are negligible given the thermometer precision of 0.2 °C. Furthermore, slightly higher temperatures can be expected for cloacal measurements because taking the measurement requires handling of the animal, which may increase its body temperature, and because the cloaca is closer to the side of the body that is in contact with the ground, and thus typically least exposed to heat loss.

### Temperature dependency of resting heart rate

All procedures have been approved by my institution’s animal care and use committee (SIUC IACUC permit# 14-011) and by the Department of public health and social services, Canton of Ticino, Switzerland. I measured resting heart rate as a proxy for aerobic metabolism and standard metabolic rate (Green, 2011). Although heart rate may not always be directly related to fitness, it is easier to measure than other physiological and demographic traits, and can provide useful information to model the response of vulnerable species to climate change. I measured resting heart rate with the Buddy device (Avitronics, Inc., Cornwall, England). This device, developed for bird breeders and the poultry industry, accurately measures heart rate in amphibians by shining an infrared beam onto the skin and by detecting small distortions caused by heart beats (Aubret, 2013; Aubret, Tort & Blanvillain, 2013).

Salamanders were captured at the study site during December 2013. Captured individuals were immediately transferred to individual terraria, where they were maintained under natural light conditions (dark:light cycle 15:30/8:30), fed ad libitum and acclimatized at field temperatures (ranging from 0 to 8 °C during the study period) during 4–6 days. Following acclimatization, each individual was exposed to four different temperatures: from 3–6 °C, 8–11 °C, 13–16 °C and 18–21 °C. Salamanders were exposed during 8 h prior to measurement of heart rate, and the order of temperature exposure was randomized among individuals. Salamanders were placed on top of the sensor and left there until the device produced a stable heart rate reading, usually within 30 s. At the time of readout, I measured *T*_*b*_ with the infrared camera. After the experiments, all salamanders were released at point of capture.

I examined the relationship between heart rate and temperature via linear mixed effects-models, with temperature and increasingly higher powers of temperature as fixed effects, and individual as a random effect to account for any differences among salamanders, using the ‘lme4’ package in R (R Core Team, 2015). I selected the best model predicting variation of resting heart rate as function of temperature on the basis of the lowest Akaike Information Criterion values corrected for sample size (AICc; Burnham & Anderson, 2002), change in AICc (ΔAICc) between the best candidate model and all other models, and the models’ AIC weights (*w*AIC, higher weight indicates better model). I then derived the temperature of peak heart rate performance, and the temperature range corresponding to a linear increase of heart rate with temperature. I extracted coefficients of the linear regression over such temperature range, and calculated fitted values over the temperature range from 1 to 20 °C (the “expected” heart rate, i.e., the rate that would be expected in absence of thermoregulatory costs). Finally, I calculated the ratio of the value fitted by the polynomial model over the value fitted by linear regression. This ratio, expressed as percentage, indicates whether the salamander’s aerobic metabolism is operating above (metabolic compensation) or below (metabolic depression) metabolism expected in absence of costs associated with low and high body temperatures.

I calculated average heart rate over an entire season for the periods of 1988–2014 (hourly temperatures available, *n* = 301192 measurements, Lugano weather station maintained by MeteoSwiss, Bern, Switzerland, and located 15 km SW of the study site, at the same elevation), as well as 1935–1939 and 1973–2014 (three temperature measurements per day: the minimum temperature, 18h00 and 6h00; no night temperature data were available for other time periods). Each season started on 7 September and ended on 24 May (days when at least one salamander was found during visits from 1989 to 2014). Furthermore, heart rate was only calculated for 1 < *T*_*a*_ < 16 °C, because no salamanders were observed beyond this temperature range, and from 18h00 to 6h00, because salamanders are nocturnal.

In order to quantify the effect of metabolic compensation at low temperatures, I used two calculation methods: (1) resting heart rate calculated from the fitted polynomial curve, and (2) expected heart rate assuming that no compensation occurs at low temperatures (i.e., by fitting the linear regression obtained in the linear portion of the performance curve). Heart rates were computed for each measurement, averaged across days and then averaged across months. Finally, monthly averages were corrected for frequency of field-active salamanders during each month (i.e., number of salamanders found during a given month across years divided by 1,343, the total number of salamanders found during the entire study period), and corrected monthly values were summed across months from September to May to produce the seasonal average heart rate. All statistical analyses were performed with R (R Core Team, 2015).

## Results

Body temperatures of field-active salamanders ranged from 0.4 to 8.7 °C (*n* = 228), and averaged 5.12 ± 0.17 °C in December, 2.77 ± 0.18 °C in January, and 6.65 ± 0.22 °C in March. Some salamanders were either much warmer (*T*_*b*_ = 8.33 °C warmer than snow temperature) or much colder (*T*_*b*_ = 4.66 °C colder than leaf litter) than substrate temperatures. Two salamanders were active on the snow when first sighted (Figs. 1A and 1B). Body temperatures were slightly colder than air temperature, on average −0.15 ± 0.07 °C (*n* = 218, *t* = − 2.06, *P* = 0.04), and colder than substrate temperatures, on average −0.35 ± 0.09 °C (*n* = 218, *t* = − 6.61, *P* < 0.001). Salamanders employed a variety of thigmothermic thermoregulatory behaviors, such as aggregating around a spring and immersion in spring water (Fig. 1C), retreating to underground burrows (Fig. 1D) and positioning on bedrock that was warmer than the top soil or leaf litter (Figs. 1E and 1F).

The salamanders’ behavior reflected their metabolic compensation when operating at low temperatures (Fig. 3). Over the study period of 26 years, most salamanders (62%) were active on the ground in cold and wet weather, and whereas a significant proportion (32%) was also active in warm and wet weather, no salamander was observed at temperatures above 16 °C. Most field-active salamanders were found during December (42.2%) and preceding months (September: 11.8%, October: 10.3%).

**Figure 2:**
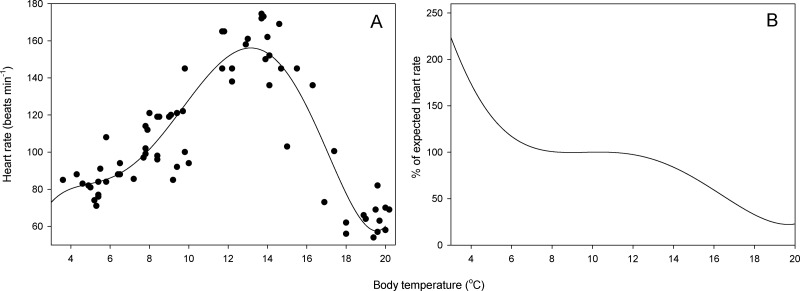
Relationship between heart rate and temperature. (A) Resting heart rate as a function of body temperature in *S. salamandra* (*n* = 16 salamanders * 4 temperature measurements). (B) Percent change in resting heart rate with respect to null expected heart rate (i.e., resting heart rate expected in the absence of physiological costs of maintaining metabolism across a range of temperatures; see text for details).

Resting heart rate ranged from 54 to 174 min^−1^ (Fig. 2A). The highest heart rate was measured at a body temperature of 13.7 °C. A 5th-rank polynomial curve best fit the observed data (*HR* = − 66.26 + 101.57*T* − 26.70*T*^2^ + 3.26*T*^3^ − 0.17*T*^4^ + 0.03*T*^5^; Table 1, *R*^2^ = 0.84, *p* < 0.001) and predicted maximum heart rate at 13.1 °C. Salamanders exhibited metabolic depression above 10.8 °C and at 16.6 °C, heart rate was only 50% of expected value (Fig. 2B). In contrast, metabolic compensation occurred at temperatures below 8.0 °C, and heart rate at 3 °C was 224% the expected heart rate (Fig. 2B).

**Figure 3:**
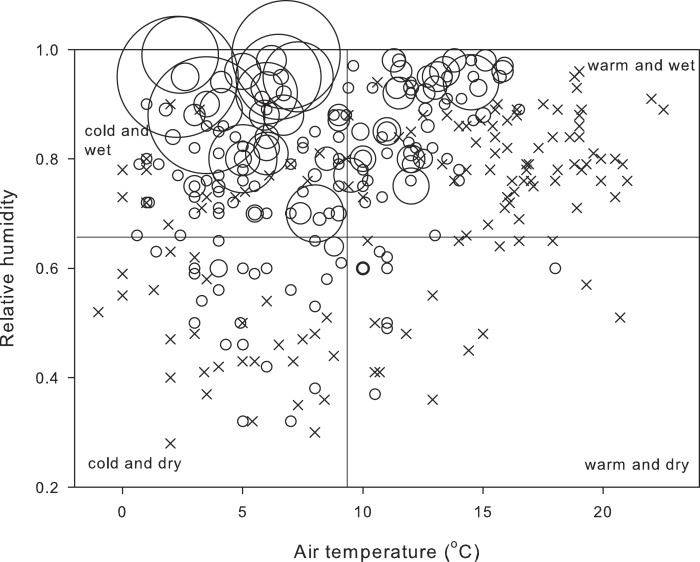
Number of field-active salamanders under different conditions of temperature and humidity. Number of field-active salamanders (*n* = 1287 salamanders) and air temperature and relative humidity during 322 nocturnal visits from 9 October 1989 to 5 January 2016. Size of dots is proportional to the number of field-active salamanders, and crosses represent nights with no salamanders. Cut-off values of the four panes for air temperature (9.3 °C) and relative humidity (0.65) are the midpoint of the range of temperature and relative humidity at which at least one salamander was found active on the ground.

**Table 1:** AICc scores of models predicting resting heart rate as a function of temperature. Akaike information criterion (AICc) scores corrected for sample size for mixed-effects models of resting heart rate as a function of temperature (T) and higher powers of T, where *k* is the number of parameters, Δ(AICc) is the change in AICc with respect to the best candidate model (in bold) and *w*(AICc) is the AICc weight.

Model variables	*k*	AICc	Δ(AICc)	*w*(AICc)
Intercept only	3	530.81	88.45	0
T	4	533.09	90.73	0
T + T2	5	473.54	31.18	0
T + T2 + T3	6	468.42	26.06	0
T + T2 + T3 + T4	7	450.78	8.42	0.02
**T**+**T2**+**T3**+**T4**+**T5**	**8**	**442.36**	**0**	**0.80**
T + T2 + T3 + T4 + T5 + T6	9	445.27	2.91	0.19

**Figure 4:**
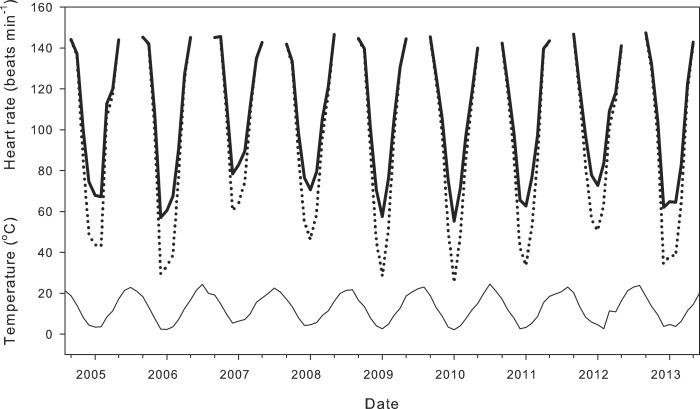
Effect of metabolic compensation on estimated daily average heart rate. Air temperature (thin line) and estimated daily average heart rate of field-active salamanders with (black line) and without (dotted line) compensation at low temperatures for the seasons from September 2004 to May 2014.

**Figure 5:**
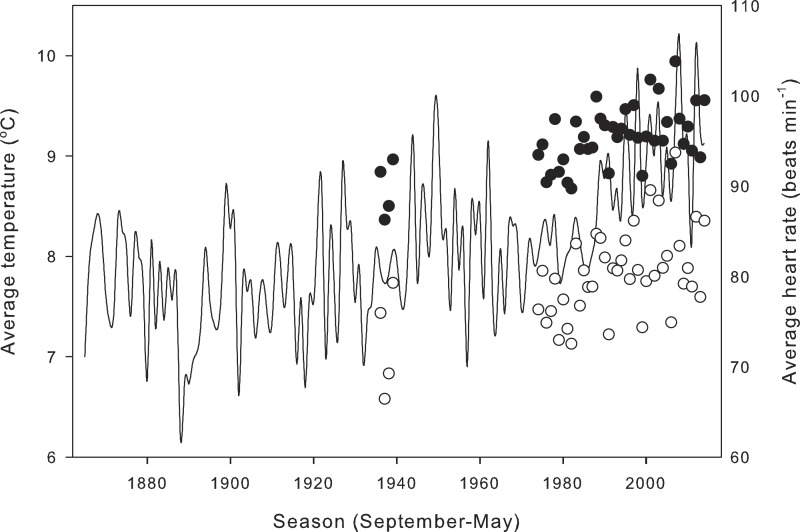
Effect of metabolic compensation on estimated seasonal heart rate. Average estimated seasonal heart rate of field-active salamanders with (black circles) and without (white circles) compensation at low temperatures, and seasonal average temperatures (line) at the closest meteorological station from 1865 to 2014.

Compensation of heart rate at low temperatures had significant effects on calculations of daily average heart rate for field-active salamanders (Fig. 4). Assuming a salamander was active on any day from September 1988 to May 2014, compensation at low temperatures inflated daily average heart rate by 11.00 ± 0.66 min^−1^ over a season (21.4 ± 1.7% difference from expected heart rate), and by up to 30 min^−1^ (118.3%) for end December, a period when salamander activity peaks. Seasonal average heart rate increased from 1935 to 2014 (Fig. 5) with (HR = 0.11*year −121.82; *R*^2^ = 0.36, *p* < 0.01) or without (HR = 0.15*year −218.43; *R*^2^ = 0.33, *p* < 0.01) taking into account compensation at low temperatures. Seasonal average heart rate with compensation was 15.24 ± 0.28 min^−1^ (19.3 ± 0.5%) higher than heart rate calculated without compensation (*t* = 54.9, *p* ≪ 0.001). Calculation method had no effect on the rate of increase (ANCOVA, *P* = 0.247 for the interaction term of year*calculation method).

## Discussion

My study documents that salamanders are behaviorally active at low temperatures, around 5 °C during late fall, and as low as 1 °C during winter. In contrast, salamanders are not field-active during summer and more generally when temperatures exceed 16 °C. Data collected over a period of 26 years show the consistency of these activity patterns. Infrared images of field-active salamanders show that opportunities to behaviorally avoid freezing temperatures are limited to thigmothermy and evasion by immersion in water or retreat underground. Resting heart rate is sensitive to temperature, causing metabolic depression at temperatures above 11 °C, and metabolic compensation at temperatures below 8 °C. Thus, salamanders operating at temperatures below 8 °C are able to maintain a higher metabolic rate than the rate expected in absence of compensation. Given that salamanders are field-active at low temperatures, my discussion will emphasize the implications of metabolic compensation for predicting the vulnerability of salamanders to global warming.

Salamanders have limited opportunities to escape freezing temperatures during cold and wet nights. Infrared images show some salamanders can maintain body temperatures considerably higher than substrate and air temperatures during nights with near-freezing temperatures. Effective freezing avoidance strategies seem to require retreat to underground burrows and immersion in spring water or fast-flowing water. As soon as salamanders are active on the ground, however, their body temperature is likely to quickly approach equilibrium with substrate and air temperatures, because opportunities of heat exchange through conduction are limited by the scarcity of suitable sources of heat above ground. Thus, it is not surprising that body temperatures of field-active salamanders did not differ greatly from air temperatures. The small difference of 0.15 ± 0.07 °C between these two measurements validates the use of air temperatures recorded by weather stations for modeling daily and seasonal average heart rates.

Physiological measurements provide a plausible explanation for summer inactivity in these salamanders, because resting heart rate sharply decreases (metabolic depression) as temperatures rise above 15 °C (Fig. 2). My field activity data collected over 26 years corroborates the finding that such sharp decrease deters behavioral activity, because no salamander has been found active at air temperatures above 16 °C. Therefore, measurement of heart rate appears to be a good proxy of temperature tolerance, or at least tolerance that is behaviorally and ecologically relevant.

These findings highlight the salamanders’ vulnerability to projected temperature increases (CH2014-Impacts, 2014). If climate warming continues unabated, temperatures in early or late months when salamanders are currently active (September and May) will exceed 16 °C and will force salamanders to remain underground. Climatic models project a nearly twofold increase in the number of “summer days,” and increasing frequency of “tropical nights” for the lower parts of Switzerland for the period to 2085 in non-intervention scenarios of the Intergovernmental Panel on Climate Change (scenarios A1B and A2, www.ipcc.ch; CH2014-Impacts, 2014). Therefore, duration of the activity season for salamanders will be reduced. Alternatively, increasing winter temperatures (CH2014-Impacts, 2014) might increase opportunities for salamanders to become active during winter periods that were previously of freezing or sub-freezing temperatures. These winter periods, often from mid-January to end February, are usually short and do not push salamanders into torpor. The opportunity to extend activity during winter could initially offset the reduction in duration of the activity season, an effect that will disappear once all winter temperatures exceed sub-freezing temperatures. Then salamanders will need to operate, forage, reproduce and grow at higher temperatures, thus requiring higher caloric intake, and over a shorter period of time (meaning the rate of required caloric intake will also be greater) than they presently do.

The metabolic compensation at low temperatures that can be inferred from observed variation in resting heart rate has important implications for understanding the vulnerability of a temperate species to climate warming. First, as shown herein, including such compensation greatly affects calculations of average heart rate and, by extension, standard metabolic rate and energy requirements. The differences in average heart rate between the model including compensation and the model excluding compensation were highest for the period of the year when salamander activity peaks (December). Therefore, current estimates of standard metabolic rates that do not account for compensation at low temperatures might underestimate energetic demands of salamanders and other organisms that exhibit similar enhanced metabolic performance at low temperatures.

Underestimation will be especially pronounced for temperate ectotherms such as salamanders that conduct a predominantly fossorial life and that favor cold temperatures for behavioral activity. A fossorial lifestyle shields these animals from extreme high temperatures, at which changes in metabolism will be greatest. In my study, salamanders were not active at temperatures above 16 °C and aestivated during the warmest months from June to August. During these months salamanders remained in underground burrows where temperatures are constant and moderate (∼8–12 °C). The greatest source of variation in metabolism thus comes from periods of high activity, namely fall and winter months when salamanders are experiencing low temperatures and when compensation of heart rate is likely to occur.

There are several ecological implications of underestimating energetic demands. The first one is that climate warming will have a greater effect on energetic needs, meaning that salamanders will experience a greater need to forage and ingest prey than expected from models that exclude compensation at low temperatures. My results suggest that from 1935 to 2014, the seasonal average heart rate of salamanders increased by ∼8%, assuming that salamanders have not shifted their seasonal activity patterns. The increase is particularly pronounced over the past three decades (Fig. 5). Estimates from meta-analysis of large datasets indicate up to 20% increase in metabolic rate over the past 20 years (Seebacher, White & Franklin, 2015). Metabolic rate of ectotherms varies non-linearly with temperature, and increases faster at warm temperatures (West, Brown & Enquist, 1997). Although compensation mechanisms at low temperatures might not have as great an effect on metabolism as high temperatures (Dillon, Wang & Huey, 2010), my results show these compensation mechanisms are important when calculations of heart or metabolic rate are made in context of the ecological, diel, and seasonal niche of behavioral activity of a temperate organism.

Increased energetic demands of ectotherms exposed to warmer climates may result in reduced body size, because thermally stressed organisms are forced to allocate more energy to maintenance (resting and activity metabolism, immunity) and reproduction so that less energy is available for growth. Indeed, body sizes of salamanders and frogs have shrunk over periods as short as 23 years (Caruso et al., 2014; Narins & Meenderink, 2014). Similarly, a wide range of organisms from plants to marine invertebrates, insects, fish and birds decreased in size in response to experimental warming and to recent climate change (reviewed by Sheridan & Bickford, 2011).

Another implication of my results is that to understand salamanders’ vulnerability to climate warming, it is important to consider what occurs at low temperatures as much as what occurs at high temperatures. In addition to its relevance for the organisms’ energetic budget, activity at low temperature might offer ecological advantages to organisms that display similar compensation mechanisms for ecologically important traits. For example, a higher than expected heart rate at low temperature in salamanders might correlate with increased predation success, and thus facilitate energy intake at times of highest behavioral activity. Increasing fall and early winter temperatures will not only increase the overall energetic budget of these salamanders, but might also force salamanders to forsake temperatures at which they exhibit metabolic compensation (i.e., if temperatures exceed 8 °C), possibly corresponding to conditions of optimal ecological performance.

Metabolic compensation at low temperatures has been reported from several high latitude (Gaston et al., 2009; Scholander et al., 1953) and high elevation organisms (Jacobsen & Brodersen, 2008). More generally, it has been proposed that such compensation is adaptive in organisms that live in cold climates (i.e., the Metabolic Cold Adaptation Hypothesis, Bozinovic, Calosi & Spicer, 2011). The idea is that an organism that can maintain high metabolism at low temperatures can compensate for the short period available to complete reproduction, development, or growth. However, the generality of this hypothesis is challenged by studies reporting decreased metabolic rates in populations of ectotherms living at high latitudes compared with conspecific populations living at low latitudes (Angilletta, 2001; Lardies, Catalan & Bozinovic, 2004; Peck, 2002). Independently of whether compensation at low temperatures is adaptive and related to the geographic distribution of organisms, several ectotherms are able to maintain higher than expected metabolic rates at low temperatures, and this compensation has implications for quantifying the effects of climate warming on metabolism.

In conclusion, my study shows the importance of considering a species performance breadth over the entire range of environmental temperatures, and specifically temperatures that are behaviorally and ecologically most relevant. Although there is a strong rationale for emphasizing physiological responses at high temperatures (i.e., in tropical environments), the physiological and ecological traits of cold tolerant species require an examination of responses at low temperatures. Metabolic compensation thus can become relevant to understand the organism’s vulnerability to climate change even if the absolute change in metabolism is much smaller than the change caused by high temperatures.

## Supplemental Information

10.7717/peerj.2072/supp-1 Supplemental Information 1 Datasets and link to video

## Additional Information and Declarations

### Data Availability

The following information was supplied regarding data availability: The raw data has been supplied as Supplemental Information.
