# Supplementary material for: Ecological implications of metabolic compensation at low temperatures in salamanders
Source: PeerJ. 2016 May 24;4:e2072. doi: 10.7717/peerj.2072 (PMC4888306; doi:10.7717/peerj.2072)
Supplement: Supplemental Information 1 [file peerj-04-2072-s001.docx]

Supplementary file (video). Video and infrared thermography of salamander (*Salamandra salamandra*) walking on snow:

<https://youtu.be/C1ydrE2q3NQ>

[
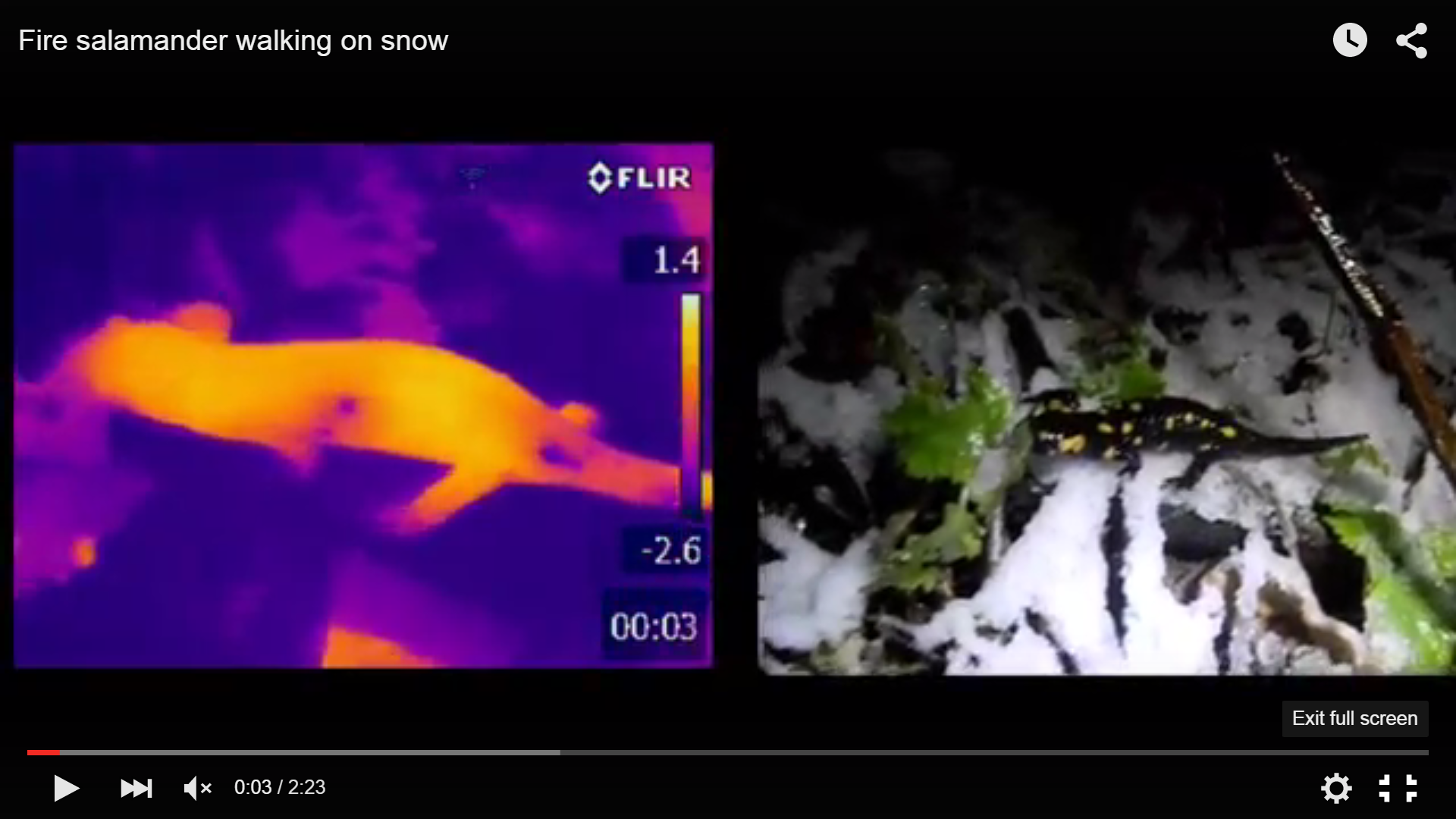
](https://youtu.be/C1ydrE2q3NQ)

Dataset 1. Heart rate measurements

ind temp HR fitted 5th order

2.13 1 7.9 112 100.50987

2.13 1 12.9 158 152.09443

2.13 1 18 62 83.46256

2.13 1 6.5 94 88.06364

74.13 2 9.4 92 119.47414

74.13 2 5.4 84 83.59441

74.13 2 7.2 85.5 93.41752

74.13 2 12.2 145 149.63791

74.13 2 19.6 57 62.96488

69.13 3 7.8 114 99.40386

69.13 3 5.3 71 83.422

69.13 3 14.1 152 148.96777

69.13 3 19.7 63 62.80959

68.13 4 7.8 102 99.40386

68.13 4 5.2 74 83.28546

68.13 4 15.5 145 132.97309

75.13 5 9 119 77.81879

75.13 5 5 81 114.13596

75.13 5 14 162 83.11522

75.13 5 19.4 54 149.6052

71.13 6 14.1 136 63.80264

71.13 6 5.8 84 148.96777

71.13 6 20 70 84.66147

71.13 6 10 94 63.52472

70.13 7 9.4 121 127.4552

70.13 7 5.4 77 119.47414

70.13 7 13.8 173 83.59441

70.13 7 17.4 100.5 150.67153

81.13 8 8.4 98 95.80868

81.13 8 5.5 91 106.42095

81.13 8 14.7 145 83.80373

81.13 8 12.2 138 143.69261

81.13 8 19 64 149.63791

78.13 9 8.5 119 67.30971

78.13 9 5.4 84 107.66832

78.13 9 16.3 136 83.59441

78.13 9 19.6 82 118.70604

73.13 10 8.4 119 62.96488

73.13 10 5.4 76 106.42095

73.13 10 11.7 165 83.59441

73.13 10 16.9 73 146.22805

80.13 11 9.1 120 106.43591

80.13 11 7.8 99 115.46266

80.13 11 9.7 122 99.40386

80.13 11 20 58 123.49028

80.13 11 8 121 63.52472

80.13 11 6.4 88 101.64329

80.13 11 13.7 174.5 87.45594

72.13 12 13 161 151.1007

72.13 12 9.8 100 152.20123

72.13 12 4.3 88 124.8204

72.13 12 11.8 165 83.4326

72.13 12 19.5 69 147.00911

66.13 13 9.2 85 63.2993

66.13 13 15 103 116.79591

66.13 13 13.7 172 140.14471

66.13 13 20.2 69 151.1007

66.13 13 4.6 83 65.09652

3.13 14 5.8 108 83.14471

3.13 14 13.9 150 84.66147

3.13 14 18.9 66 150.17309

3.13 14 3.6 85 68.51328

65.13 15 9.8 145 84.58892

65.13 15 14.6 169 124.8204

65.13 15 18 56 144.74263

65.13 15 4.9 82 83.46256

1.13 16 6.5 88 83.07877

1.13 16 8.4 96 88.06364

1.13 16 7.7 97 106.42095

1.13 16 20.7 61 98.32667

1.13 16 11.7 145 73.54964

Dataset 2. IR camera measurements

Ref Date Time Ta RH SVL Sex IR files Tbody average Tsub

1.13 19-Dec-13 21:30 2.3 95 100 female 62-65 1.7 2.0

2.13 19-Dec-13 21:45 2.3 95 89.3 male 66-67 3.2 3.5

3.13 19-Dec-13 21:55 2.3 95 100 female 68-71 1.1 1.2

4.13 19-Dec-13 22:00 2.3 95 100 female 72-75 1.7 1.5

5.13 19-Dec-13 22:08 2.3 95 99.5 male 78-79 1.0 1.2

6.13 19-Dec-13 22:08 2.3 95 85.6 male 80-83 0.7 0.8

7.13 19-Dec-13 22:20 2.3 95 98 male 84-85 0.9 1.6

8.13 19-Dec-13 22:30 2.3 95 83 male 86-87 1.5 1.7

9.13 19-Dec-13 22:45 2.3 95 53 juv 88-89 3.7 4.8

NA 19-Dec-13 23:05 2.3 95 90-95 2.7 3.4

NA 19-Dec-13 23:05 2.3 95 96-99 2.6 3.3

10.13 19-Dec-13 23:05 2.3 95 96.8 female 100-103 2.7 3.6

NA 19-Dec-13 23:06 2.3 95 female NA

11.13 19-Dec-13 23:22 2.3 95 83.2 male 104-105 3.2 5.0

12.13 19-Dec-13 23:22 2.3 95 84 female 106-107 2.1 2.8

13.13 19-Dec-13 23:35 2.3 95 55 juv 110-111 2.1 2.8

14.13 19-Dec-13 23:35 2.3 95 84.8 female 108-109 2.3 2.8

15.13 19-Dec-13 23:45 2.3 95 51.2 juv 112-113 3.8 3.6

16.13 19-Dec-13 23:45 2.3 95 57.5 juv 114-115 3.0 3.6

17.13 19-Dec-13 23:52 2.3 95 87.3 male 116-119 3.0 3.4

18.13 20-Dec-13 0:16 2.3 95 92.5 male 124-125 2.3 2.1

19.13 20-Dec-13 0:20 2.3 95 94 male 120-121 3.3 3.8

20.13 20-Dec-13 0:20 2.3 95 53.4 juv 122-123 2.7 3.7

21.13 20-Dec-13 0:35 2.3 95 95.5 female 126-127 #DIV/0!

22.13 20-Dec-13 0:40 2.3 95 88.5 female 128-129 0.4 1.2

23.13 20-Dec-13 0:40 2.3 95 96 female 130-133 1.3 1.5

24.13 20-Dec-13 0:40 2.3 95 83.3 male 134-135 1.9 2.5

25.13 23-Dec-13 20:10 6.5 95 84.4 female 182-183 6.4 6.4

26.13 23-Dec-13 20:15 6.5 95 92 male 184-185 6.6 6.2

27.13 23-Dec-13 20:30 6.5 95 91 female 186-187 7.0 7.2

28.13 23-Dec-13 20:43 6.5 95 95 male 188-191 6.8 6.6

29.13 23-Dec-13 20:44 6.5 95 92.5 female 192-193 6.6 6.6

30.13 23-Dec-13 20:45 6.5 95 100 male 194-195 7.1 7.2

31.13 23-Dec-13 20:53 6.5 95 90.5 female 196-197 6.9 7.0

32.13 23-Dec-13 21:05 6.5 95 99 female 198-199 7.5 7.8

33.13 23-Dec-13 21:17 6.5 95 81 juv 200-201 6.7 6.9

34.13 23-Dec-13 21:20 6.5 95 86 male 202-203 6.6 6.5

35.13 23-Dec-13 21:22 6.5 95 74 juv 204-205 7.0 6.6

36.13 23-Dec-13 21:25 6.5 95 92.6 male 206-207 6.6 6.3

37.13 23-Dec-13 21:35 6.5 95 91.5 female 208-209 4.6 4.4

38.13 23-Dec-13 21:40 6.5 95 80 female NA #DIV/0!

39.13 23-Dec-13 21:50 6.5 95 95.5 male 210-211 7.1 7.0

40.13 23-Dec-13 21:55 6.5 95 101 female 212-213 6.4 6.2

41.13 23-Dec-13 22:00 6.5 95 100 female 214-215 5.9 5.9

42.13 23-Dec-13 22:15 6.5 95 94 male 216-217 3.9 3.6

43.13 23-Dec-13 22:18 6.5 95 107 female 218-219 6.9 7.2

44.13 23-Dec-13 22:20 6.5 95 86.5 male 220-221 6.3 6.4

45.13 23-Dec-13 22:30 6.5 95 92.5 male 222-223 6.7 6.3

46.13 23-Dec-13 22:35 6.5 95 95 female 224-225 6.3 6.1

47.13 23-Dec-13 22:50 6.5 95 87 female 226-227 6.6 6.4

48.13 23-Dec-13 22:50 6.5 95 87 male 228-229 6.4 6.8

49.13 23-Dec-13 22:50 6.5 95 100 male 230-231 6.6 6.8

50.13 23-Dec-13 22:55 6.5 95 56 juv 232-233 6.8 7.2

51.13 23-Dec-13 23:05 6.5 95 88 male 234-235 6.2 6.5

52.13 23-Dec-13 23:08 6.5 95 82 male 236-237 6.5 6.5

53.13 23-Dec-13 23:10 6.5 95 84 male 238-239 6.3 6.1

54.13 23-Dec-13 23:25 6.5 95 55 juv 240-241 6.6 6.4

55.13 23-Dec-13 23:30 6.5 95 71 juv 242-243 4.1 3.9

56.13 23-Dec-13 23:45 6.5 95 96 female 244-245 6.9 6.8

57.13 23-Dec-13 23:46 6.5 95 97 female 246-247 6.1 5.8

58.13 23-Dec-13 23:50 6.5 95 93.5 female NA #DIV/0!

59.13 23-Dec-13 23:55 6.5 95 88 male 248-249 4.1 4.2

60.13 24-Dec-13 0:00 6.5 95 96 male 250-251 7.3 7.9

61.13 24-Dec-13 0:05 6.5 95 67 juv NA #DIV/0!

62.13 24-Dec-13 0:06 6.5 95 53.7 juv NA #DIV/0!

63.13 24-Dec-13 0:18 6.5 95 99 female 252-255 7.4 7.9

64.13 24-Dec-13 0:26 6.5 95 71.5 juv NA #DIV/0!

65.13 24-Dec-13 0:45 6.5 95 86.3 male NA #DIV/0!

66.13 24-Dec-13 0:45 6.5 95 63 juv NA #DIV/0!

76.13 28-Dec-13 21:50 4.8 90 95.5 female 312-313 5.2 5.8

77.13 28-Dec-13 21:55 4.8 90 95 female 314-315 5.1 5.0

78.13 28-Dec-13 22:00 4.8 90 95 male 316-319 5.1 5.2

79.13 28-Dec-13 22:11 4.8 90 90 female 320-323 3.4 3.7

80.13 28-Dec-13 22:18 4.8 90 81 324-325 4.5 4.4

81.13 28-Dec-13 22:24 4.8 90 72.5 female 326-327 4.4 3.9

67.13 28-Dec-13 21:24 4.8 90 118 female 282-285 5.9 6.7

68.13 28-Dec-13 21:26 4.8 90 99.5 female 286-289 7.2 5.6

69.13 28-Dec-13 21:35 4.8 90 82.7 male 298-299 5.1 4.9

70.13 28-Dec-13 21:32 4.8 90 88 female 296-297 4.5 4.4

71.13 28-Dec-13 21:30 4.8 90 97.5 female 292-295 7.2 6.6

72.13 28-Dec-13 21:46 4.8 90 64.5 juv 301-311 5.0 5.2

73.13 28-Dec-13 21:14 4.8 90 97 female 276-277 5.2 5.4

74.13 28-Dec-13 21:21 4.8 90 98.5 female 278-279 5.2 6.3

75.13 28-Dec-13 21:40 4.8 90 104 male 300-309 5.7 7.3

82.13 2-Jan-14 22:20 2 90 74.5 male 346-350 4.1 8.2

83.13 2-Jan-14 22:25 2 90 50.4 Bufo bufo 351-357 #DIV/0!

84.13 2-Jan-14 22:35 2 90 65 juv 358-369 2.2 4.0

85.13 2-Jan-14 22:35 2 90 65.5 juv 370-383 3.9 5.4

86.13 2-Jan-14 22:50 2 90 98 male 384-391 2.0 3.2

87.13 2-Jan-14 22:50 2 90 102 female 392-393 6.2 8.8

88.13 2-Jan-14 23:10 2 90 72.5 male 394-411 3.9 5.4

NA 4-Jan-14 23:15 4.5 90 36.5 juv 499-505 4.8 6.1

NA 4-Jan-14 23:10 4.5 90 89.5 male 412-498,506 8.1 -0.2

NA 4-Jan-14 23:59 4.5 90 102 male 52#-541 5.4 6.3

NA 4-Jan-14 23:55 4.5 90 68.5 juv 542-547 8.8 10.2

NA 5-Jan-14 0:10 4.5 90 95 male 548-563 5.8 7.2

NA 5-Jan-14 0:10 4.5 90 103 female 548-563 6.5 7.2

NA 5-Jan-14 0:45 4.5 90 90 female 564-583 1.8 1.7

228.14 11-Mar-14 7.4 70 1533-1536 7.8 9.7

229.14 11-Mar-14 7.4 70 1537-1540 6.5 6.9

230.14 11-Mar-14 7.4 70 1541-1544 5.2 6.0

231.14 11-Mar-14 7.4 70 1545-1548 5.4 6.8

232.14 11-Mar-14 7.4 70 1548-1553 5.5 6.0

233.14 11-Mar-14 7.4 70 1555-1560 7.1 8.6

NA 11-Mar-14 7.4 70 1561-1573 6.0 6.7

234.14 7.4 70 NA

235.14 11-Mar-14 7.4 70 NA

236.14 11-Mar-14 7.4 70 1571-1576 6.2

237.14 11-Mar-14 7.4 70 1577-1590 7.5 8.8

238.14 11-Mar-14 7.4 70 1594-1599 6.1 6.4

239.14 11-Mar-14 7.4 70 1602-1605 5.0 6.4

240.14 14-Mar-14 8.8 64 1606-1609 6.8 7.2

241.14 14-Mar-14 8.8 64 1610-1613 7.3 7.5

242.14 14-Mar-14 8.8 64 1614-1617 6.8 7.4

243.14 14-Mar-14 8.8 64 1618-1625 7.4 8.8

244.14 14-Mar-14 8.8 64 1626-1629 8.1 8.9

245.14 14-Mar-14 8.8 64 1630-1635 8.1 9.0

246.14 14-Mar-14 8.8 64 1636-1639 6.3 7.1

247.14 14-Mar-14 8.8 64 1640-1643 7.0 9.2

248.14 14-Mar-14 8.8 64 1644-1647 7.0 8.0

1513.14 14-Dec-14 21:55 7.3 95 63.5 juv 2866-2867 6.8 6.5

1514.14 14-Dec-14 22:00 7.3 95 79 male 2868-2869 5.8 5.6

1515.14 14-Dec-14 22:10 7.3 95 56.5 juv 2872-2873 6.0 5.6

1516.14 14-Dec-14 22:15 7.3 95 62.3 juv 2874-2875 7.0 6.9

1517.14 14-Dec-14 22:22 7.3 95 41.5 juv 2876-2877 6.5 6.5

1518.14 14-Dec-14 22:35 7.3 95 92 male 2878-2879 5.0 5.2

1519.14 14-Dec-14 22:36 7.3 95 90 male 2884-2885 8.0 7.9

1520.14 14-Dec-14 22:45 7.3 95 90.5 female 2880-2881 7.6 7.6

1521.14 14-Dec-14 23:00 7.3 95 96 female 2886-2887 6.8 6.9

1522.14 14-Dec-14 23:05 7.3 95 96.5 male 2888-2889 7.1 7.1

1523.14 14-Dec-14 23:08 7.3 95 55 juv 2890-2891 7.0 7.1

1524.14 14-Dec-14 23:30 7.3 95 93 female 2892-2893 7.1 7.4

1525.14 14-Dec-14 23:31 7.3 95 79.5 female 2894-2895 6.9 6.9

1526.14 14-Dec-14 23:40 7.3 95 70.5 juv 2896-2897 7.0 7.0

1527.14 14-Dec-14 23:55 7.3 95 97 female 2898-2901 8.6 9.7

1528.14 14-Dec-14 0:00 7.3 95 95 male 2902-2905 7.7 8.2

1529.14 14-Dec-14 0:08 7.3 95 88.5 male 2906-2909 7.7 9.5

1530.14 14-Dec-14 0:12 7.3 95 81 male 2910-2911 6.9 7.0

1531.14 14-Dec-14 0:20 7.3 95 101 female 2912-2913 6.4 6.4

1532.14 14-Dec-14 0:30 7.3 95 88.5 male 2914-2915 7.2 7.2

1533.14 14-Dec-14 0:34 7.3 95 35.5 juv 2916-2917 7.7 7.9

1534.14 14-Dec-14 0:45 7.3 95 76.5 male 2918-2919 6.9 6.8

1535.14 14-Dec-14 0:48 7.3 95 92 female 2920-2921 6.9 6.9

1536.14 14-Dec-14 1:00 7.3 95 72 juv 2922-2923 7.5 7.4

1537.14 14-Dec-14 1:08 7.3 95 73.5 male 2924-2925

1538.14 14-Dec-14 1:10 7.3 95 91 female 2926-2927 7.0 6.9

1539.14 14-Dec-14 1:20 7.3 95 68 male 2928-2929 6.7 6.6

1540.14 14-Dec-14 1:22 7.3 95 97 male 2930-2931 8.0 8.4

1541.14 14-Dec-14 1:30 7.3 95 76.5 male 2932-2933

1542.14 14-Dec-14 1:43 7.3 95 86 female 2934-2935 6.6 6.6

1543.14 14-Dec-14 1:44 7.3 95 65 female 2936-2937 7.6 7.8

1544.14 14-Dec-14 1:46 7.3 95 48 juv 2938-2939 6.4 6.6

1545.14 14-Dec-14 2:00 7.3 95 92 female 2940-2941 6.5 6.5

1546.14 14-Dec-14 2:15 7.3 95 93 male 2948-2949 6.7 6.8

Dataset 3. Seasonal variation in number of salamanders, temperature and relative humidity from 1989 to 2014.

Date Time T[°C] H[%] N salamanders

m/d/y start hh.mm T[°C] H[%]

10/9/1989 17.45 17.45-18.00 10.5 50% 0

10/27/1989 18.00 18.00-18.30 4

10/29/1989 8

11/3/1989 8

11/9/1989 19.00 19.00-19.30 11 50% 1

11/10/1989 19.00 19.00-19.30 10 60% 4

11/11/1989 19.00 19.00-19.30 6 60% 2

11/17/1989 19.00 19.00-19.30 9 70% 7

11/19/1989 19.00 19.00-19.30 11 60% 2

11/22/1989 16.30 16.30-17.30 12 75% 17

12/15/1989 19.00 19.00-20.00 8 70% 27

12/16/1989 16.30 16.30-17.30 8 85% 2

12/17/1989 16.30 16.30-17.30 11 85% 13

1/18/1990 17.45 17.45-18.45 4 60% 8

1/26/1990 13.00 13.00-13.30 10 60% 6

1/28/1990 11.00 11.00-12.00 6 70% 4

1/29/1990 12.00 12.00-12.30 7 70% 1

2/2/1990 13.15 13.15-13.30 9 75% 5

2/14/1990 15.30 15.30-16.15 10 75% 1

4/3/1990 19.30 19.30-20.30 10 80% 8

9/29/1992 19.30 19.30-20.30 12 94% 4

9/30/1992 19.30 19.30-20.30 13.5 80% 4

10/1/1992 19.30 19.30-20.30 11 85% 9

10/2/1992 16.30 16.30-18.30 13 93% 11

10/10/1992 17.00 17.00-18.00 12.5 80% 9

10/19/1992 19.30 19.30-20.30 6 89% 4

11/15/1992 19.30 19.30-20.30 5 80% 8

11/25/1992 19.30 19.30-20.30 8.5 80% 11

11/29/1992 20.00 20.00-21.00 5 78% 1

12/1/1992 20.00 20.00-21.00 6 84% 11

12/2/1992 18.00 18.00-19.00 6 92% 29

12/5/1992 19.30 19.30-20.30 7 56% 3

12/7/1992 17.30 17.30-18.30 1 90% 1

12/12/1992 19.30 19.30-20.30 3 70% 2

12/18/1992 19.30 19.30-20.30 1 78% 0

12/22/1992 19.30 19.30-20.00 3 62% 0

12/26/1992 19.30 19.30-20.30 1 72% 0

12/28/1992 19.30 19.30-20.30 0 59% 0

1/2/1993 19.30 19.30-20.30 -1 52% 0

1/7/1993 19.30 19.30-20.30 5.5 70% 8

1/9/1993 19.30 19.30-20.30 4 65% 2

1/11/1993 19.30 19.30-20.30 5.5 70% 6

1/13/1993 19.30 19.30-20.30 6 81% 20

1/16/1993 19.30 19.30-20.30 1 80% 5

1/18/1993 19.30 19.30-20.30 3 82% 1

1/19/1993 19.30 19.30-20.30 3 73% 1

1/21/1993 19.30 19.30-20.30 3 59% 2

1/23/1993 19.30 19.30-20.30 4 74% 2

1/25/1993 19.30 19.30-20.30 7 32% 2

1/28/1993 19.30 19.30-20.30 3 75% 2

1/29/1993 17.30 17.30-18.30 5 60% 1

1/30/1993 16.00 16.00-17.00 4 81% 1

1/30/1993 19.45 19.45-20.45 4 75% 6

2/2/1993 19.30 19.30-20.30 3.5 37% 0

2/6/1993 19.30 19.30-20.30 5 43% 0

2/9/1993 19.30 19.30-20.30 4 70% 2

2/13/1993 19.30 19.30-20.30 5 46% 1

2/16/1993 19.30 19.30-20.30 3 50% 1

2/21/1993 19.30 19.30-20.30 4 42% 0

2/24/1993 19.30 19.30-20.30 2 28% 0

2/27/1993 19.30 19.30-20.30 3.5 76% 2

2/28/1993 11.30 11.30-12.30 1.5 79% 1

3/2/1993 18.00 18.00-19.30 3 75% 10

3/7/1993 19.30 19.30-20.30 8 30% 0

3/10/1993 20.00 20.00-20.45 6 54% 0

3/14/1993 20.00 20.00-21.00 8 53% 1

3/21/1993 20.00 20.00-21.00 13 66% 4

3/31/1993 21.15 21.15-22.00 8 48% 0

4/3/1993 21.15 21.15-22.30 8 77% 6

4/11/1993 19.00 19.00-20.00 9 80% 3

4/25/1993 19.00 19.00-20.00 9 84% 3

5/4/1993 21.30 21.30-22.30 14 65% 0

5/13/1993 21.30 21.30-22.30 12 85% 0

6/10/1993 15.00 15.00-15.30 16 82% 0

6/10/1993 22.30 22.30-23.00 15.5 81% 0

6/22/1993 22.30 22.30-23.00 15.5 84% 0

7/10/1993 22.30 22.30-23.00 16 87% 0

8/23/1993 22.30 22.30-23.00 19 84% 0

8/27/1993 21.30 21.30-22.00 14 86% 0

9/9/1993 22.00 22.00-23.00 15 88% 7

9/13/1993 22.00 22.00-23.00 18 60% 1

9/16/1993 22.00 22.00-23.00 14 78% 1

9/22/1993 21.30 21.30-22.00 18 76% 0

9/24/1993 17.30 17.30-19.15 14 76% 5

9/25/1993 17.00 17.00-18.00 14 76% 3

9/28/1993 21.45 21.45-22.45 10 78% 4

10/1/1993 21.45 21.45-23.30 12 80% 13

10/4/1993 22.00 22.00-22.45 10 78% 4

10/7/1993 18.30 18.30-19.30 11 81% 3

10/11/1993 21.30 21.30-22.30 12 81% 7

10/16/1993 21.30 21.30-22.30 10 80% 12

10/19/1993 21.30 21.30-22.30 12 79% 8

10/22/1993 21.45 21.45-22.45 7 79% 0

10/24/1993 14.00 14.00-16.00 9 79% 4

11/2/1993 22.45 22.45-24.15 9.5 77% 16

11/7/1993 20.45 20.45-22.00 9 84% 5

11/13/1993 21.00 21.00-22.00 7 79% 2

11/20/1993 21.30 21.30-22.30 2 47% 0

11/26/1993 21.30 21.30-22.30 2 63% 0

12/3/1993 21.45 21.45-22.45 4 74% 1

12/7/1993 21.30 21.30-24.00 5 80% 32

12/13/1993 20.30 20.30-21.30 4 56% 4

12/18/1993 21.00 21.00-21.30 4 71% 1

12/22/1993 21.00 21.00-21.30 3 48% 0

12/28/1993 21.00 21.00-21.30 2 40% 0

1/6/1994 16.30 16.30-17.00 0 78% 0

1/8/1994 16.00 16.00-16.30 4 85% 1

1/10/1994 20.45 20.45-22.00 6 82% 12

1/15/1994 20.45 20.45-21.45 4 77% 2

1/20/1994 21.00 21.00-22.00 5 32% 1

1/23/1994 21.00 21.00-22.00 3 60% 3

1/27/1994 20.45 20.45-21.45 3.5 58% 0

1/31/1994 20.45 20.45-21.45 6 42% 1

2/4/1994 20.45 20.45-21.45 5 80% 16

2/9/1994 20.45 20.45-21.45 5 50% 0

2/13/1994 20.45 20.45-21.45 1 80% 0

2/16/1994 20.45 20.45-21.45 0 55% 0

2/18/1994 21.00 21.00-21.30 0 73% 0

2/22/1994 21.00 21.00-22.00 3 73% 1

2/26/1994 21.15 21.15-22.30 5 76% 5

2/28/1994 21.00 21.00-22.30 3 88% 10

3/5/1994 21.30 21.30-22.30 5.5 59% 2

3/7/1994 21.30 21.30-22.30 8 65% 5

3/10/1994 21.30 21.30-22.30 11 62% 4

3/14/1994 21.30 21.30-22.30 11 49% 1

3/18/1994 21.30 21.30-22.30 7.5 47% 0

3/26/1994 21.45 21.45-22.45 10.5 37% 1

4/1/1994 17.30 17.30-19.30 9 88% 10

4/8/1994 22.00 22.00-23.00 8 38% 1

4/16/1994 22.30 22.30-24.15 9 87% 10

4/22/1994 21.45 21.45-22.45 12 76% 1

4/29/1994 22.00 22.00-23.00 16.5 65% 0

5/6/1994 22.15 22.15-23.15 15 48% 0

5/11/1994 22.30 22.30-23.30 12 92% 2

5/20/1994 22.30 22.30-23.30 12.5 88% 0

5/30/1994 22.15 22.15-23.15 15.5 89% 0

6/11/1994 22.40 22.40-23.40 13.5 88% 0

6/18/1994 23.30 23.30-00.30 19 88% 0

6/25/1994 23.15 23.15-24.00 20.5 73% 0

7/2/1994 23.30 23.30-00.30 22 91% 0

7/10/1994 23.30 23.30-00.30 21 76% 0

7/14/1994 23.30 23.30-00.30 19 96% 0

7/23/1994 23.45 23.45-00.30 20.8 79% 0

7/30/1994 23.45 23.45-00.30 20.5 80% 0

8/6/1994 23.30 23.30-00.30 18.8 95% 0

8/13/1994 21.00 24.00-00.45 18.6 84% 0

8/20/1994 23.45 23.45-00.30 19.9 76% 0

8/27/1994 24.00 24.00-00.45 18.8 79% 0

9/3/1994 23.30 23.30-00.30 16.4 89% 0

9/8/1994 22.30 22.30-00.30 15.8 96% 10

9/10/1994 23.30 23.30-00.30 15.6 90% 0

9/14/1994 12.30 12.30-14.15 15.1 98% 10

9/16/1994 22.30 22.30-00.30 11.3 98% 11

9/22/1994 22.30 22.30-00.15 13.8 98% 11

9/24/1994 22.30 22.30-00.30 15.9 97% 8

9/26/1994 24.00 24.00-01.00 14.8 93% 8

10/1/1994 22.30 22.30-23.30 15.7 95% 6

10/3/1994 22.00 22.00-23.30 13.4 96% 14

10/6/1994 21.30 21.30-22.30 10.2 76% 2

10/15/1994 23.00 23.00-24.00 11.5 84% 0

10/22/1994 18.15 18.15-19.30 12 93% 7

10/23/1994 21.00 21.00-22.30 10.4 93% 1

10/29/1994 23.15 23.15-00.15 7.5 83% 3

12/10/1994 22.30 22.30-23.30 6.6 95% 7

12/26/1994 22.30 22.30-23.30 1 78% 0

12/29/1994 22.00 22.00-24.00 5 96% 17

1/21/1995 1.00 01.00-01.30 2 90% 0

2/11/1995 0.00 24.00-02.00 6 93% 15

3/4/1995 23.00 23.00-23.45 4.3 46% 1

3/11/1995 22.00 22.00-23.00 5.5 75% 2

3/25/1995 23.30 23.30-00.30 8.8 88% 1

4/1/1995 23.00 23.00-23.30 14.4 45% 0

4/15/1995 22.30 22.30-23.30 8.5 51% 0

7/30/1995 23.00 23.00-24.00 19.1 89% 0

9/3/1995 21.30 21.30-22.30 15.4 86% 0

9/5/1995 22.30 22.30-23.00 14.3 66% 0

9/7/1995 22.30 22.30-23.30 13.4 90% 2

9/17/1995 21.30 21.30-22.30 14.3 86% 0

9/18/1995 22.00 22.00-23.00 14.1 91% 2

9/22/1995 22.00 22.00-22.30 13.5 91% 1

9/24/1995 21.30 21.30-22.30 11.6 96% 10

9/28/1995 22.00 22.00-23.00 12.9 55% 0

10/2/1995 10.00 10.00-11.30 12.6 92% 9

10/6/1995 22.00 22.00-23.00 14.5 98% 4

10/10/1995 23.30 23.30-00.30 16.5 89% 1

10/12/1995 23.30 23.30-00.30 14.9 87% 0

10/16/1995 23.00 23.00-23.30 13.3 79% 0

10/17/1995 10.30 10.30-11.00 12.4 80% 0

10/28/1995 22.00 22.00-23.00 10.8 73% 2

10/29/1995 10.00 10.00-12.00 9.9 85% 8

11/3/1995 22.00 22.00-23.00 5.4 32% 0

11/4/1995 12.00 12.00-12.15 7.1 43% 0

11/17/1995 23.00 23.00-24.30 8.2 69% 6

11/19/1995 15.00 15.00-15.30 7.3 35% 0

12/8/1995 22.30 22.30-01.15 4.1 89% 20

12/9/1995 22.00 22.00-22.30 3.2 89% 0

12/10/1995 15.00 15.00-15.30 4.7 73% 0

12/22/1995 22.30 22.30-00.30 5.9 88% 14

12/24/1995 16.30 16.30-17.00 6.1 77% 0

12/26/1995 15.30 15.30-17.15 6.7 92% 7

12/30/1995 16.00 16.00-16.30 0.6 66% 1

1/3/1996 15.00 15.00-16.00 8.5 58% 1

1/19/1996 22.30 22.30-23.30 1.3 56% 0

1/21/1996 14.00 14.00-14.30 5.5 59% 1

2/10/1996 21.30 21.30-22.30 1 72% 2

2/11/1996 16.00 16.00-16.30 3.4 41% 0

2/14/1996 22.00 22.00-22.45 3.3 54% 1

3/5/1996 22.30 22.30-23.30 1.4 63% 1

3/7/1996 14.00 14.00-14.30 3.5 86% 1

3/7/1996 23.30 23.30-1.30 2.6 95% 13

3/15/1996 22.00 22.00-23.00 7.5 74% 4

3/17/1996 4.00 4.00-5.15 6.2 98% 14

4/2/1996 4.30 4.30-5.30 4.2 86% 2

4/7/1996 0.30 00.30-1.30 10.2 65% 0

5/3/1996 22.30 22.30-23.30 9.4 88% 0

5/18/1996 23.00 23.00-24.00 14.6 95% 1

7/6/1996 0.00 24.00-00.45 16.4 88% 0

7/21/1996 4.30 4.30-5.00 16.7 76% 0

7/28/1996 0.00 00.00-00.30 17.3 82% 0

10/18/1996 21.45 21.45-23.15 9.3 88% 3

10/25/1996 22.30 22.30-00.30 11.5 92% 15

10/27/1996 15.00 15.00-15.30 11.9 83% 2

11/15/1996 23.30 23.30-00.30 9.6 97% 3

11/29/1996 23.45 23.45-00.15 1.9 68% 0

12/22/1996 22.00 22.00-00.00 6.7 88% 19

12/25/1996 22.30 22.30-23.45 5.8 91% 7

1/2/1997 0.00 00.00-1.00 1.1 72% 4

1/3/1997 22.00 22.00-00.00 2.1 84% 7

1/31/1997 23.00 23.00-23.45 3.3 71% 0

2/18/1997 22.15 22.15-23.00 5.1 74% 0

2/23/1997 23.00 23.00-23.45 8.2 81% 0

2/24/1997 22.00 22.00-22.45 7.7 76% 0

3/8/1997 20.00 20.00-21.00 9.4 75% 0

3/29/1997 22.30 22.30-23.15 8.4 36% 0

3/31/1997 23.45 23.45-24.15 8.8 44% 0

4/2/1997 0.30 00.30-1.00 11.8 48% 0

4/5/1997 23.30 23.30-00.00 10.7 41% 0

4/26/1997 0.15 00.15-1.15 12.6 89% 6

4/27/1997 17.20 17.20-17.40 14 1

4/27/1997 23.00 23.00-00.00 9.3 80% 0

5/17/1997 0.30 00.30-1.00 16.8 79% 0

7/10/1997 23.30 23.30-00.00 15.3 78% 0

7/15/1997 23.00 23.00-00.00 19.3 57% 0

7/19/1997 0.30 00.30-1.00 16.5 69% 0

8/28/1997 0.15 00.15-1.00 18.9 93% 0

8/29/1997 0.00 00.00-00.30 13.8 76% 0

9/1/1997 23.15 23.15-00.00 19 86% 0

9/2/1997 22.15 22.15-22.45 19.9 80% 0

9/3/1997 22.00 22.00-22.30 19.1 78% 0

9/8/1997 1.00 1.00-1.30 18.9 71% 0

9/8/1997 22.45 22.45-23.15 19.3 79% 0

9/9/1997 23.00 23.00-23.45 20.7 51% 0

9/10/1997 23.45 23.45-00.30 17.9 65% 0

9/11/1997 12.30 12.30-13.30 18.1 78% 0

9/12/1997 14.30 14.30-15.00 18.2 0

9/12/1997 22.00 22.00-22.45 18.1 89% 0

9/13/1997 22.15 22.15-23.00 16.8 78% 0

9/15/1997 23.45 23.45-00.30 16.1 72% 0

9/16/1997 23.30 23.30-00.00 15.2 68% 0

9/17/1997 23.30 23.30-00.00 15.9 71% 0

9/18/1997 22.30 22.30-23.15 16.3 74% 0

9/19/1997 23.45 23.45-00.15 15.7 64% 0

9/20/1997 23.30 23.30-00.15 17.1 75% 0

9/21/1997 23.15 23.15-00.00 16.9 79% 0

9/22/1997 23.15 23.15-23.45 17 76% 0

9/23/1997 23.15 23.15-23.45 16.9 79% 0

9/24/1997 23.30 23.30-00.15 16.2 76% 0

9/25/1997 22.30 22.30-23.15 16 73% 0

10/21/1997 23.00 23.00-1.00 13.1 95% 14

10/22/1997 15.00 15.00-16.00 0

10/22/1997 22.30 22.30-23.45 12.7 86% 6

10/23/1997 23.15 23.15-00.15 12.9 82% 5

10/24/1997 23.45 23.45-00.15 12.9 36% 0

10/25/1997 9.00 9.00-9.15 8 48% 0

10/26/1997 21.15 21.15-21.45 10.1 72% 1

10/27/1997 21.00 21.00-22.00 10.7 63% 2

2/1/1998 22.12 22.15-22.45 2.3 77% 3

2/7/1998 22.45 22.45-23.45 2.4 66% 2

2/8/1998 16.00 16.00-17.00 6.5 46% 0

2/14/1998 1.45 1.45-2.30 9.1 61% 2

2/14/1998 22.30 22.30-23.30 8.6 70% 1

2/15/1998 12.00 12.00-13.00 10 73% 0

3/13/1998 17.30 17.30-18.00 0

3/14/1998 0.30 00.30-1.00 5.5 43% 0

3/14/1998 23.00 23.00-23.30 10.5 41% 0

3/24/1998 23.00 23.00-23.30 4.9 50% 1

4/4/1998 1.30 1.30-2.30 11.5 98% 6

4/4/1998 23.30 23.30-00.30 9.4 93% 2

4/17/1998 23.15 22.15-23.15 7.5 82% 1

4/25/1998 23.45 23.45-00.30 14.3 78% 0

5/24/1998 23.45 23.45-00.30 12.2 82% 2

6/9/1998 16.00 16.00-16.30 0

7/20/1998 13.00 13.00-13.30 0

8/9/1998 22.30 22.30-23.30 22.5 89% 0

9/26/1998 22.30 22.30-23.30 13.5 95% 4

9/27/1998 23.00 23.00-23.45 14.6 87% 1

10/5/1998 0.00 0.00-0.45 10.8 84% 2

10/24/1999 0.30 0.30-1.30 11.4 93% 10

10/31/1999 0.30 00.30-1.30 12.7 95% 10

12/9/1999 22.00 22.00-0.30 3.5 90% 12

12/16/2011 22.45 22.45-01.45 3.5 88% 55

9/25/2012 21.45 21.45-00.00 14.5 94% 26

12/25/2012 23.00 23.00-01.20 6.8 99% 51

12/19/2013 21.30 21.30-01.30 2.3 95% 57

12/23/2013 20.00 20.00-00.40 6.5 95% 40

3/11/2014 22.30 22.30-00.30 7.4 70% 10

3/13/2014 22.40 22.40-00.30 8.8 64% 9

8/9/2014 23.00 23.00-23.45 19.6 81% 0

8/12/2014 23.30 23.30-24.15 17.9 84% 0

12/14/2014 21.45 21.45-02.15 7.3 95% 33

12/27/2014 23.15 23.15-00.30 0.7 79% 1
